# Nano-Indentation Response of Ultrahigh Molecular Weight Polyethylene (UHMWPE): A Detailed Analysis

**DOI:** 10.3390/polym12040795

**Published:** 2020-04-02

**Authors:** Tanveer Iqbal, S. S. Camargo, Saima Yasin, Ujala Farooq, Ahmad Shakeel

**Affiliations:** 1 Department of Chemical, Polymer & Composite Materials Engineering, University of Engineering & Technology, KSK Campus, Lahore 54890, Pakistan; tanveer@uet.edu.pk (T.I.); drsaima@uet.edu.pk (S.Y.); 2 Department of Materials and Metallurgical Engineering, University Federal do Rio De Janeiro, 21941-901 Rio de Janeiro, Brazil; camargo@metalmat.ufrj.br; 3 Faculty of Aerospace Engineering, Department of Aerospace Structures and Materials, Delft University of Technology, Kluyverweg 1, 2629 HS Delft, The Netherlands; U.Farooq@tudelft.nl; 4 Faculty of Civil Engineering and Geosciences, Department of Hydraulic Engineering, Delft University of Technology, Stevinweg 1, 2628 CN Delft, The Netherlands

**Keywords:** nano-indentation, ultrahigh molecular weight polyethylene, modulus, hardness, creep

## Abstract

Nano-indentation, a depth sensing technique, is a useful and exciting tool to investigate the surface mechanical properties of a wide range of materials, particularly polymers. Knowledge of the influence of experimental conditions employed during nano-indentation on the resultant nano-mechanical response is very important for the successful design of engineering components with appropriate surface properties. In this work, nano-indentation experiments were carried out by selecting various values of frequency, amplitude, contact depth, strain rate, holding time, and peak load. The results showed a significant effect of amplitude, frequency, and strain rate on the hardness and modulus of the considered polymer, ultrahigh molecular weight polyethylene (UHMWPE). Load-displacement curves showed a shift towards the lower indentation depths along with an increase in peak load by increasing the indentation amplitude or strain rate. The results also revealed the strong dependence of hardness and modulus on the holding time. The experimental data of creep depth as a function of holding time was successfully fitted with a logarithmic creep model (R^2^ ≥ 0.98). In order to remove the creeping effect and the nose problem, recommended holding times were proposed for the investigated polymer as a function of different applied loads.

## 1. Introduction

Ultrahigh molecular weight polyethylene (UHMWPE) belongs to the polyethylene group of polymers with a repeating unit, [C_2_H_4_]_n_, where n represents the degree of polymerization. Solid samples of UHMWPE, a linear, semi crystalline polymer, consist of two distinct regions, the crystalline and amorphous regions [1]. It has comparatively higher toughness, higher abrasion resistance, and lower coefficient of friction than most of the other polymers used for biomedical applications due to the existence of molecular chain enatnglements between the very long polymer chains [2]. It shows viscoplastic and viscoelastic behaviors in both laboratory and commercial applications, well above its T_g_ of −150 °C, in addition to its rate and temperature dependent mechanical behavior [3]. The mechanical characteristics of UHMWPE can also be manipulated by controlling the morphology of the crystalline regions and the % crystallinity of the polymer [4]. Due to the above mentioned properties, UHMWPE has been widely used as a biomaterial for total joint replacement and joint arthroplasty [5].

Near-to-surface material characterization has always been a challenge. The contact performance of materials is believed to be governed by the properties of the outermost surface molecular layers. Therefore, knowledge of the surface properties are important considerations for material selection and design in applications involving surface engineering and tribological contacts. The mechanical properties of the outermost surface layers have been conveniently investigated using the nano-indentation technique for the past two decades by us and a number of other researchers [6,7,8,9,10,11,12,13,14,15,16,17,18]. Nano-indentation, a non-destructive technique, is a powerful tool for analyzing the surface mechanical properties of a wide variety of materials, including polymers, metals, composites, and biological entities.

The determination of near-to-surface mechanical characteristics by conventional indentation is problematic mainly due to its measurement of residual contact area by optical methods. Optical imaging of the contact area following indentation has been considered unsuitable for viscoelastic polymers because of comparatively high and unavoidable uncertainty for small penetration depths due to recovery and visualization problems. The difficulty of determining the contact area of indentation has been essentially overcome by utilizing a contact compliance method. In this method, loading-unloading curves are obtained by applying a particular penetration (displacement) and recording the response of the material. The analysis of the resultant curves is used to provide the hardness and modulus of the tested material. Hence, this method eliminates the requirement of measuring the residual contact area of indentation [19,20]. The inevitable existence of physical imperfections in the tip geometry of a nano-indenter is another difficulty which has been found during material analysis by a nano-indentation technique [21,22]. These tip defects could be equivalent to the full indent size for lower penetration depths and might result in substantial errors in the calculated values. An analytic method to address the tip defects for polymeric materials has been proposed by Briscoe and Sebastian [22]. This method was utilized in the present work to avoid the effects of tip defects on the measured parameters.

Numerous research groups have described the application of a nano-indentation technique to investigate the surface mechanical properties of various materials [6,7,8,9,10,11,12,13,14,15,16,17,18,19,20,21,22,23,24,25,26,27,28,29,30,31]. Chakraborty et al. [23], for instance, reported the nano-mechanical characteristics of multifunctional ZnO/PMMA-based nanocomposites using a nano-indenter. The surface properties of gallium arsenide, molybdenum, aluminum, fused silica, and tungsten were also studied with the help of a nano-indentation technique [24]. The effect of strain rate on the nano-mechanical behavior of metallic and polymeric materials has been analyzed by using the normal indentation mode and a continuous stiffness indentation mode [25,26]. Zhang et al. [27] investigated the influence of indentation holding time in nano-indentation experiments on zirconia dental ceramic. The creep effect on the nano-mechanical analysis of polypropylene in response to the applied load was studied by Ngan and Tang [28]. Fasce et al. [29] reported the nano-wear and surface properties of pristine and ion-irradiated UHMWPE with the help of nano-indentation and scanning probe microscopy. The nano-mechanical properties of reinforced UHMWPE by multi-walled carbon nanotubes at varying concentrations were analyzed by Sreekanth and Kanagaraj using tensile and nano-indentation testing [5]. The results showed an optimal concentration of MWCNTs (2 wt.%) to enhance the mechanical properties of the resultant composite system. Ho et al. [30] examined the hardness and modulus of untreated and treated compression molded UHMWPE inserts used for knee replacements using a nano-indenter. The nano-mechanical behavior of polymeric nanocomposites was also studied using nano-scratching and nano-indentation methods [5,23,31]. The outcome of these studies revealed that the addition of nanomaterials improved the modulus, hardness, and coefficient of friction of the prepared composites [31]. Recently, we described the effect of acetone exposure, in relation to different exposure times, on the surface properties of poly (ether ether ketone) (PEEK) using a nano-indentation technique [10]. The results showed a decreasing trend in the surface properties of PEEK as a function of exposure time to the acetone, which was attributed to the softening of the polymer network.

Due to the strain and strain rate dependency, polymers display different nano-mechanical behaviors under varying contact conditions. Thus, the surface mechanical characteristics of polymers strongly depends on the applied contact conditions, for example the indenter loading rate (strain rate), the penetration depth (the strain), amplitude, frequency, holding time, etc. Therefore, a thorough and complete understanding of the influence of such experimental parameters on the nano-mechanical behavior of UHMWPE is required for the effective design of engineering components composed of it.

The compliance curves of polymeric materials may show a creep effect at the onset of the unloading segment during indentation. A slight increase in penetration depth has been observed at the start of the unloading segment, meaning that the material creep rate was higher than the indenter unloading rate during the initial phase of the unloading; even the highest unloading rates did not prevent this effect. The creep data can be readily distinguished by the existence of a typically round shape of the compliance data that is known as “a nose”, at the inception of the loading-unloading segment. The problem can be overcome by using a fixed hold segment at the maximum load before the subsequent unloading segment [13]. However, a detailed analysis of this creep phenomenon as a function of different holding times is required for UHMWPE to predict the appropriate value of holding time to guarantee the eradication of this undesired phenomenon and to obtain the accurate mechanical properties of the polymer.

By keeping these objectives in mind, the present study was focused on the investigation of the nano-mechanical behavior of UHMWPE as a function of various experimental variables, including frequency, amplitude, peak load, strain rate, and holding time. Moreover, the creeping effect in UHMWPE was also studied by varying the applied holding times and peak load in order to be able to predict the appropriate holding time for the considered polymer.

## 2. Experimental

**Figure 1:**
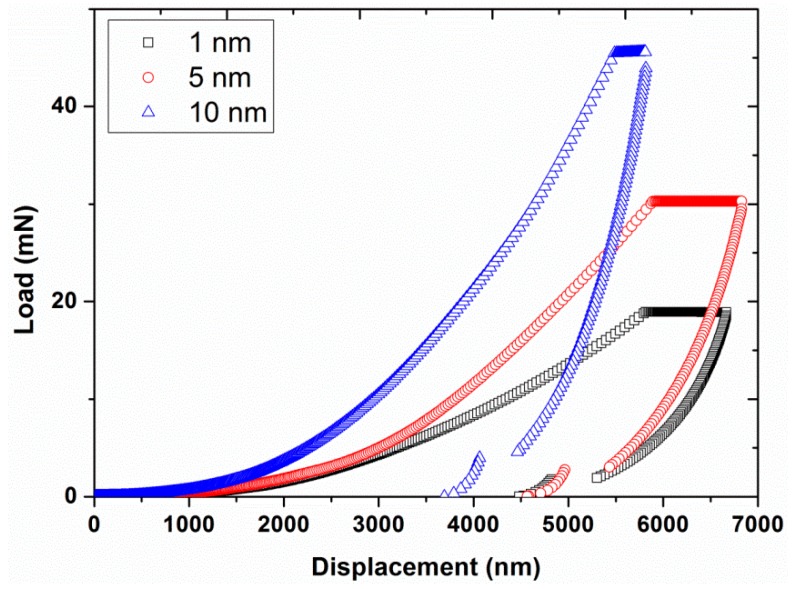
Indentation load as a function of displacement for the three different amplitudes (1, 5, and 10 nm) at a frequency of 45 Hz and strain rate of 0.05 s^−1^^.^

**Figure 2:**
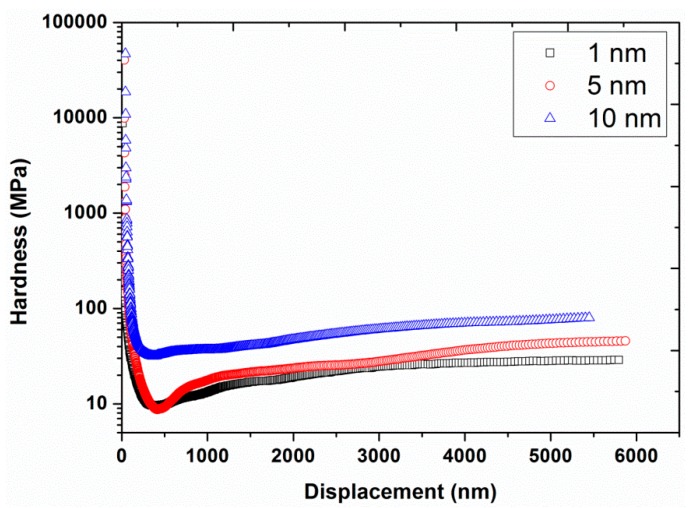
Hardness as a function of displacement for the three different amplitudes (1, 5, and 10 nm) at a frequency of 45 Hz and strain rate of 0.05 s^−1.^

**Figure 3:**
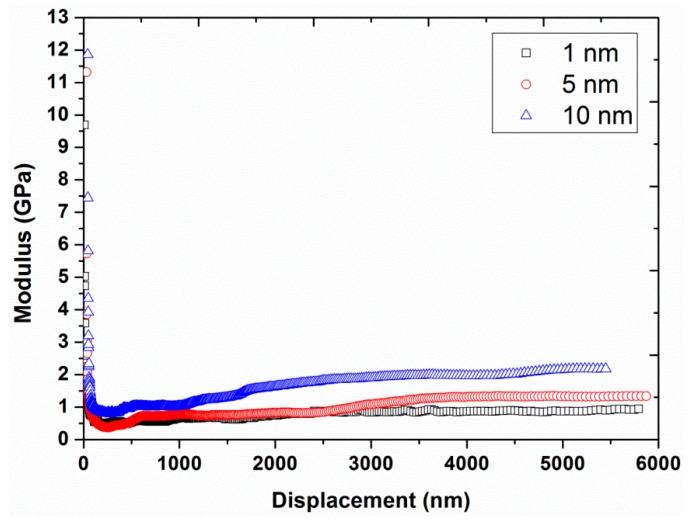
Modulus as a function of displacement for the three different amplitudes (1, 5, and 10 nm) at a frequency of 45 Hz and strain rate of 0.05 s^−1.^

Semi-crystalline ultrahigh molecular weight polyethylene (UHMWPE) sheets having 1.1 mm thickness were purchased from Goodfellow Cambridge Ltd., UK (catalogue # ET303060). The experiments were carried out without any thermal treatment of the sheets. An MTS Nano-Indenter IIs (Nano Instruments Ltd., USA) was used to perform the nano-indentation analysis of the polymeric sheets by utilizing both normal and continuous stiffness modes of indentations. The surface mechanical properties, the elastic modulus, and hardness, of the polymer were analyzed using the contact compliance mode instead of assessing the area of indentation. A three-sided pyramid Berkovich Indenter was used, which made an angle of 65.3° with the normal to the base of indenter [13]. All sides of the tip were grinded sharp, resulting in a point penetration during the nano-indentation. The tip radius of a typical Berkovich tip was approximately in the range of 100 nm. The indenter was mounted on a square indenter column but the indentation into the material has been only to the level of pyramidal geometry. Therefore, the three-sided pyramid was penetrated to all penetration depths during the indentation. The normal force on the indenter was produced using a magnetic field through an electromagnetic aluminum coil present on the top of the inner indenter tube. The MTS NANO IIs is capable of operating at a constant loading rate, a step loading, a constant displacement rate, and a constant strain rate loading. Therefore, different experimental protocols were employed to study the effect of experimental conditions on the nano-indentation response of UHMWPE, given as follows:

Experiment #1: To analyze the effect of nano-indentation amplitude, three different amplitudes, 1, 5, and 10 nm, were chosen in continuous stiffness mode (hardness and modulus values recorded continuously as the indenter was oscillated at the harmonic frequency and simultaneously continuously forced into the sample), by having a constant strain rate of 0.05 s^−1^ and a constant harmonic frequency of 45 Hz (results are presented in Figure 1, Figure 2 and Figure 3).

**Figure 4:**
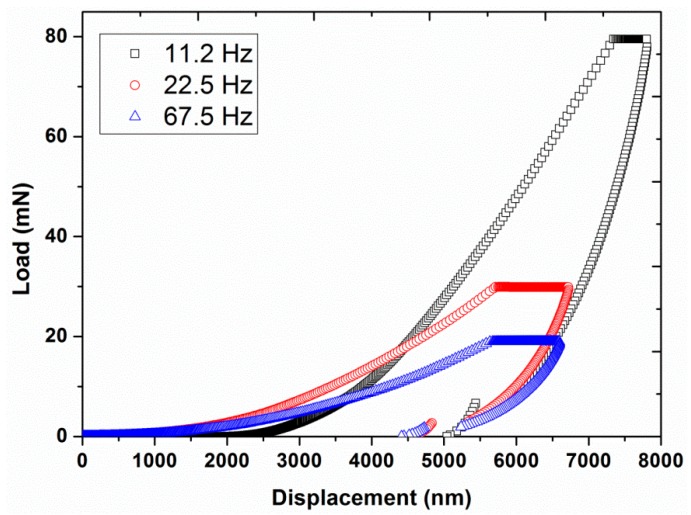
Indentation load required to yield various displacements for the three different frequencies (11.2, 22.5, and 67.5 Hz) at a vibration amplitude of 2 nm and strain rate of 0.05 s^−1.^

**Figure 5:**
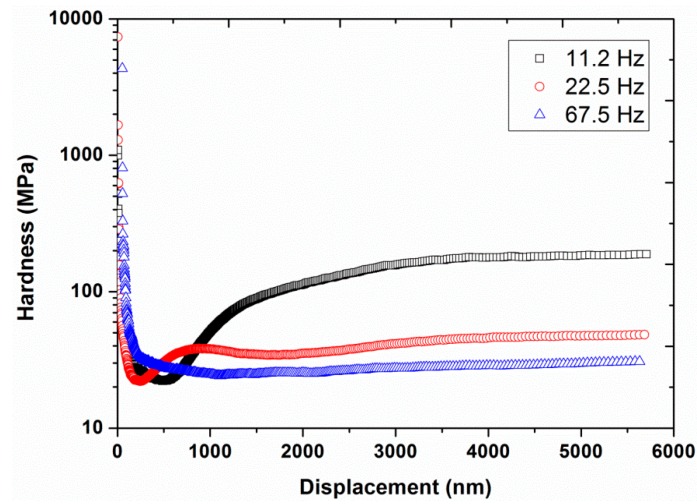
Hardness as a function of displacement for the three different frequencies (11.2, 22.5, and 67.5 Hz) at a vibration amplitude of 2 nm and strain rate of 0.05 s^−1^.

**Figure 6:**
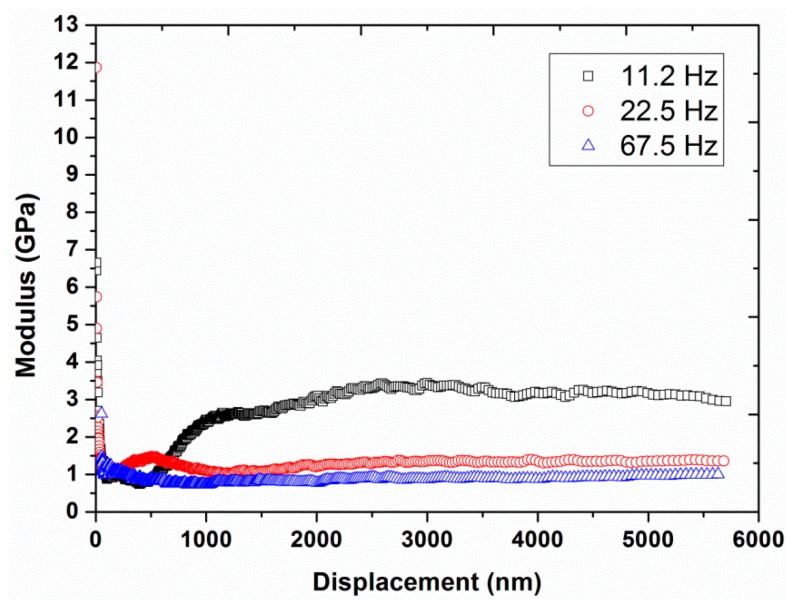
Modulus as a function of displacement for the three different frequencies (11.2, 22.5, and 67.5 Hz) at a vibration amplitude of 2 nm and strain rate of 0.05 s^−1^.

Experiment #2: To analyze the effect of harmonic frequency, three different frequencies, 11.2, 22.5, and 67.5 Hz, were utilized in continuous stiffness mode, by having a constant vibration amplitude of 2 nm and a constant strain rate of 0.05 s^−1^ (results are presented in Figure 4, Figure 5 and Figure 6).

**Figure 7:**
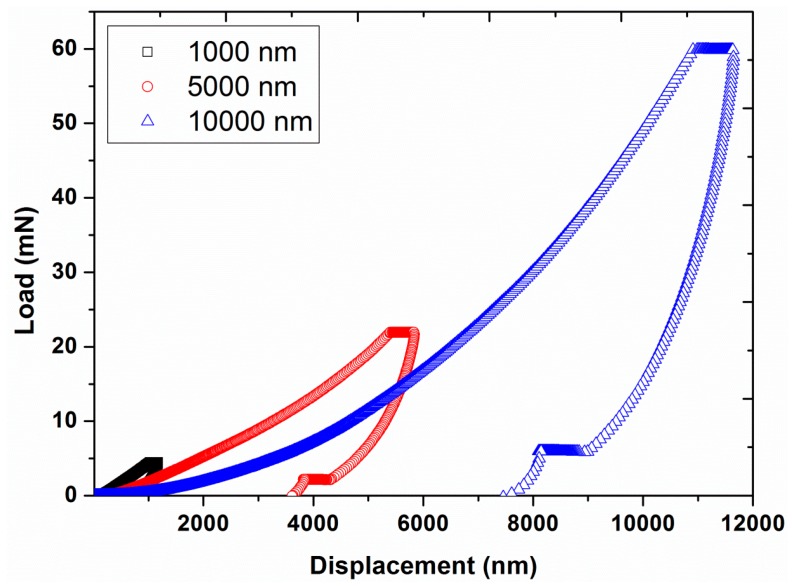
Indentation load as a function of displacement for the three different contact depths (1000, 5000, and 10000 nm) at a vibration amplitude of 2 nm and strain rate of 0.05 s^−1^.

**Figure 8:**
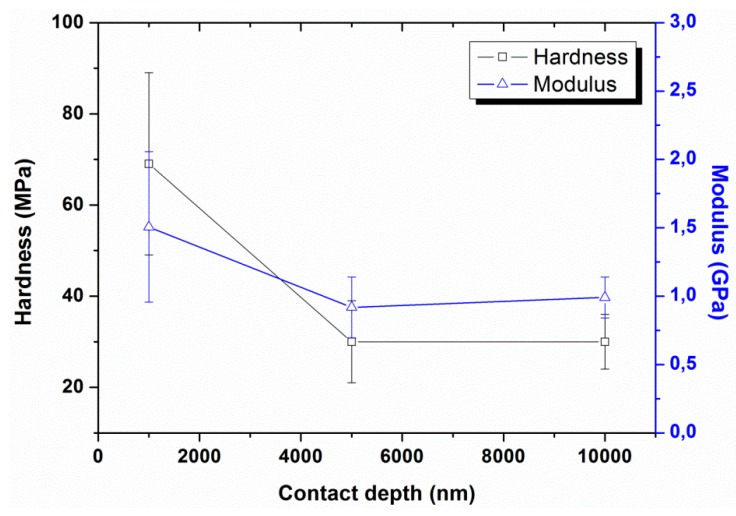
Hardness and modulus as a function of contact depth at a vibration amplitude of 2 nm and strain rate of 0.05 s^−1^. The solid lines are just a guide for the eye. Bars represent standard deviations.

Experiment #3: To study the behaviors at high indentation depth, three different indentation depths, 1000, 5000, and 10,000 nm, were used in continuous stiffness mode, by having a constant vibration amplitude of 2 nm, a constant strain rate of 0.05 s^−1^, and a constant harmonic frequency of 45 Hz (results are presented in Figure 7 and Figure 8).

**Figure 9:**
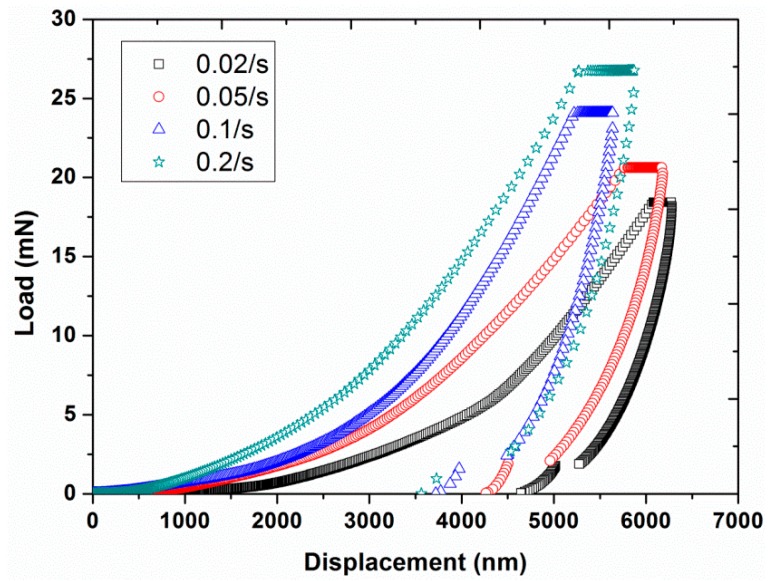
Indentation load as a function of displacement for the four different strain rates (0.02, 0.05, 0.1, and 0.2 s^−1^) at a frequency of 45 Hz and vibration amplitude of 2 nm.

**Figure 10:**
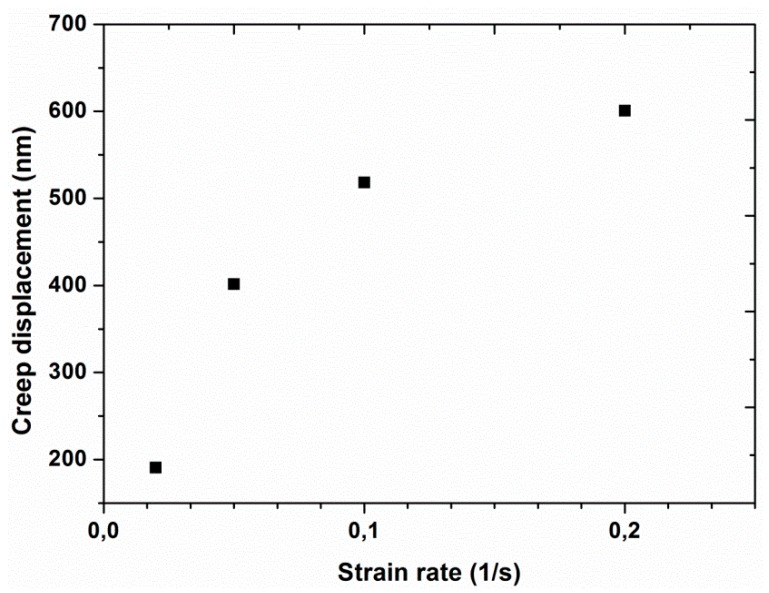
Creep displacement as a function of strain rate at a constant frequency of 45 Hz and an amplitude of 2 nm.

**Figure 11:**
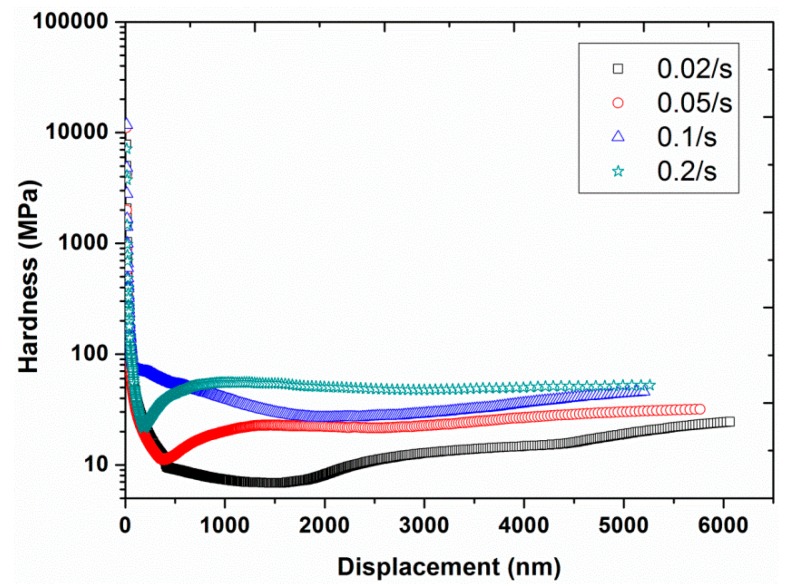
Hardness as a function of displacement for the four different strain rates (0.02, 0.05, 0.1, and 0.2 s^−1^) at a frequency of 45 Hz and vibration amplitude of 2 nm.

**Figure 12:**
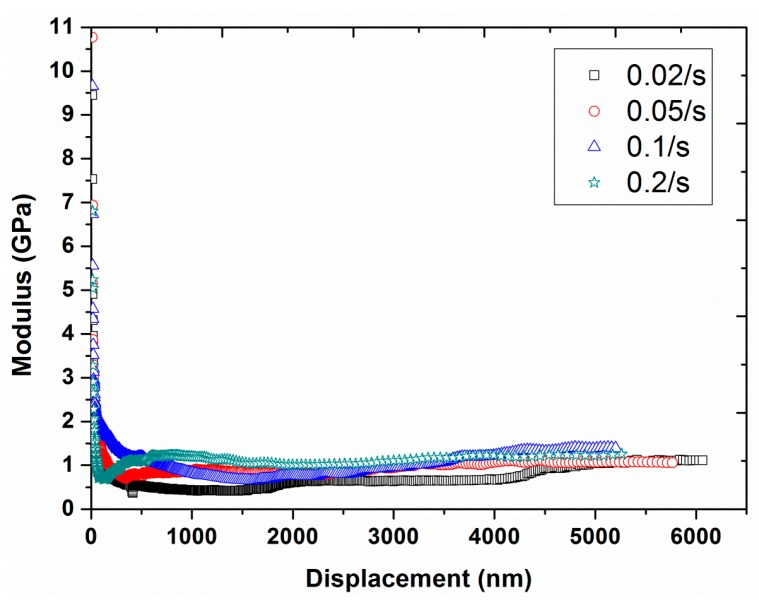
Modulus as a function of displacement for the four different strain rates (0.02, 0.05, 0.1, and 0.2 s^−1^) at a frequency of 45 Hz and vibration amplitude of 2 nm.

Experiment #4: To examine the effect of strain rate, four different strain rates, 0.02, 0.05, 0.1, and 0.2 s^−1^, were selected in continuous stiffness mode, by having a constant frequency of 45 Hz and a constant vibration amplitude of 2 nm (results are presented in Figure 9, Figure 10, Figure 11 and Figure 12).

**Figure 13:**
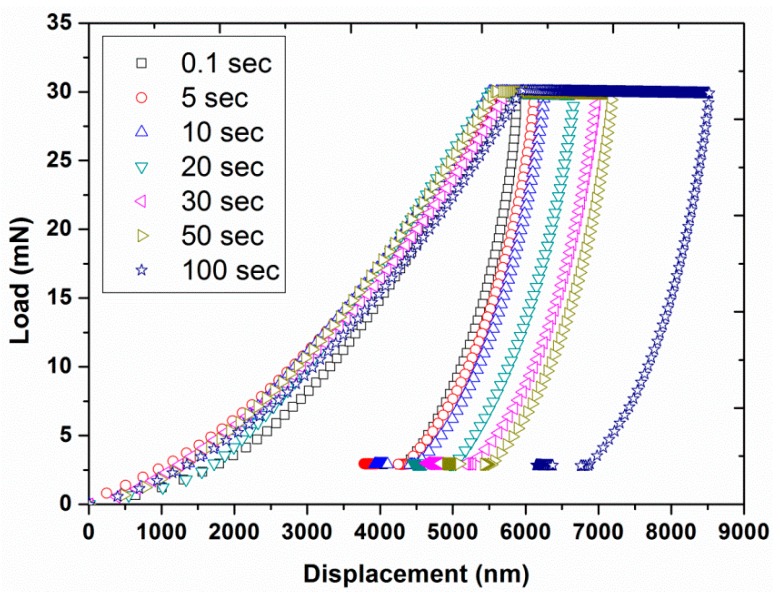
Indentation load as a function of displacement by having a peak load of 30 mN for various holding times (0.1, 5, 10, 20, 30, 50, and 100 s) with loading time of 10 s.

**Figure 14:**
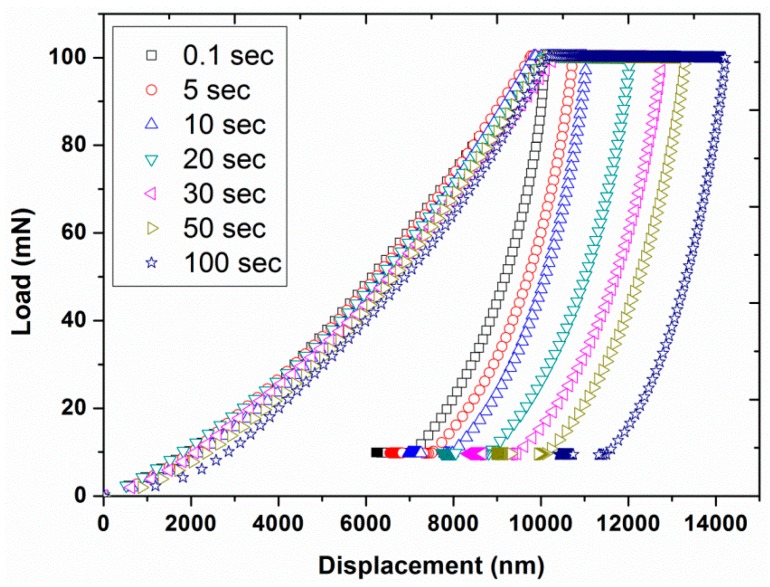
Indentation load as a function of displacement by having a peak load of 100 mN for various holding times (0.1, 5, 10, 20, 30, 50, and 100 s) with loading time of 10 s.

**Figure 15:**
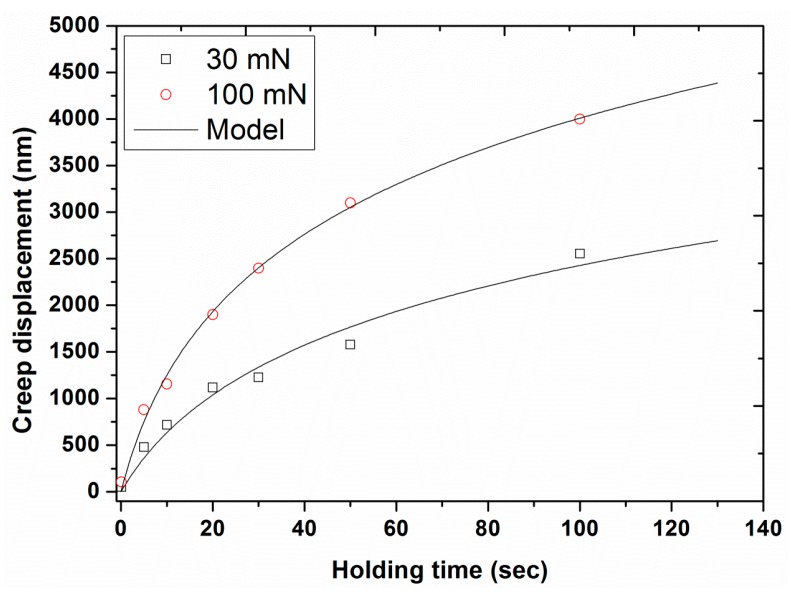
Creep displacement as a function of holding time for the two different peak loads, 30 and 100 mN. The solid line represents model fitting.

**Figure 16:**
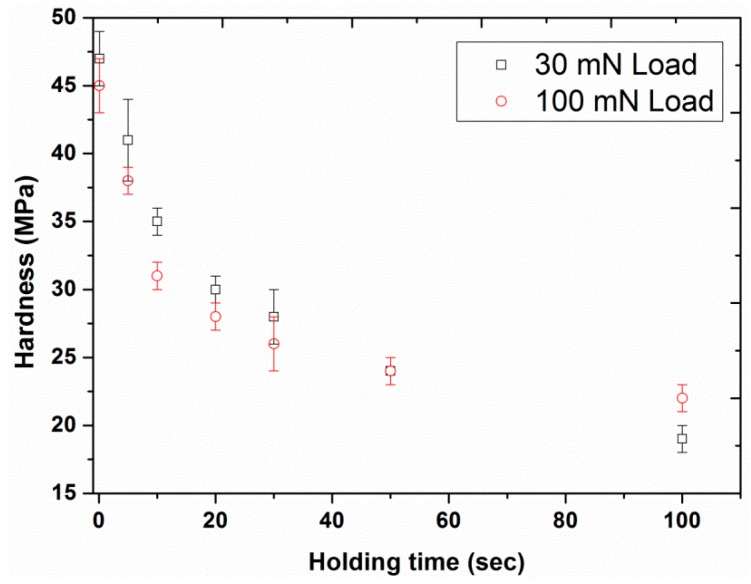
Hardness as a function of holding time for the two different peak loads, 30 and 100 mN with loading time of 10 s. Bars represent standard deviations.

**Figure 17:**
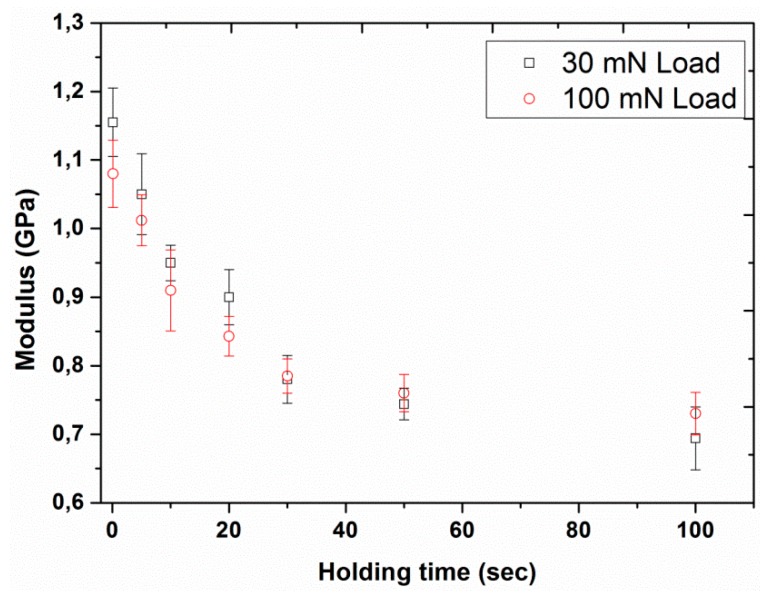
Modulus as a function of holding time for the two different peak loads, 30 and 100 mN with loading time of 10 s. Bars represent standard deviations.

**Figure 18:**
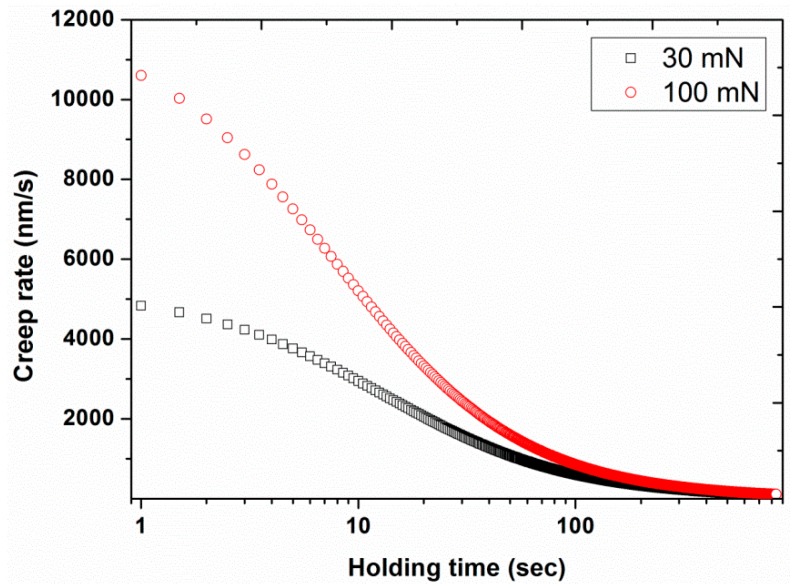
Creep rate as a function of holding time for the two peak loads, 30 and 100 mN.

Experiment #5: To assess the effect of holding time at the peak load, various holding times of 0.1, 5, 10, 20, 30, 50, and 100 s were considered in normal indentation mode, by having two different peak loads (30 and 100 mN) and a constant loading time of 10 s (results are presented in Figure 13, Figure 14, Figure 15, Figure 16, Figure 17 and Figure 18) prior to unloading.

For all the nano-indentation experiments, a hold segment was adopted after 80% unloading to account for the thermal drift. All the experiments were repeated six times and the average values are reported with the standard deviations. 

## 3. Results and Discussion

### 3.1. Effect of Amplitude, Frequency and Contact Depth 

The average load-displacement curves of UHMWPE are shown in Figure 1 for the different amplitudes at 45 Hz. The results revealed an increase in the required peak load, from 18.94 to 45.55 mN, in addition to a shift of the load-displacement curves towards the smaller indentation depths by increasing the amplitude from 1 to 10 nm. This behavior suggested a harder response of the UHMWPE at the higher indentation amplitudes. The flat line/region of the load-displacement curves represent the load hold segment that was applied to reduce the creep problem. Likewise, the hardness and modulus are presented in Figure 2 and Figure 3, respectively, as functions of the displacement for the different amplitudes. Significant decreases in hardness, and to a lesser extent, modulus values were observed at indentation displacements from 0–250 nm (i.e., indentation size effects). This peculiar behavior is suggested to be linked with the defects in the nano-indenter tip, errors in the surface estimation, and changes in the surface properties of the polymer due to the environmental conditions/processing such as oxidation of the polymer surface, exclusion of additives or impurities, etc. [13,32]. Similar results have also been reported in the literature for nano-indentation of various metals [24].

Figure 2 and Figure 3 also show a significant increase in hardness (from 27 to 70 MPa at a displacement of 4000 nm) and modulus (from 0.9 to 2.0 GPa at a displacement of 4000 nm) by increasing the vibration amplitude from 1 to 10 nm. These values of hardness and modulus are in close agreement with the already reported values of hardness (40 MPa) and modulus (1.3 GPa) of UHMWPE at 45 Hz and 1 nm [9]. In addition, the effect of indentation frequency on the load-displacement curves, hardness, and modulus of UHMWPE, at a vibration amplitude of 2 nm at 45 Hz, are shown in Figure 4, Figure 5 and Figure 6. Contrary to the effect of indentation amplitude, a decrease in peak load (from 79.52 to 18.97 mN), hardness (from 178 to 29 MPa at a displacement of 4000 nm) and modulus (from 3.2 to 0.9 GPa at a displacement of 4000 nm) was evident by increasing the frequency from 11.2 to 67.5 Hz. 

The effect of different indentation depths on the nano-mechanical properties of UHMWPE was also analyzed at 45 Hz and 1 nm and the results are presented in Figure 7 and Figure 8. The results revealed the requirement of higher peak loads in order to attain the higher displacement depths (4.53 mN for 1000 nm and 59.85 mN for 10,000 nm). A large decrease in hardness and modulus values occurred with increasing the contact depths, which we suggest, are again linked with the indentation size effects and will be explained in the next section. A similar decrease in hardness and modulus values as a function of contact depth was also reported in the literature for polymeric nanocomposite films having CdSe quantum dots as fillers [33].

### 3.2. Effect of Strain Rate 

The average load-displacement curves of UHMWPE are presented in Figure 9 for the four different strain rates of 0.02, 0.05, 0.1, and 0.2 s^−1^ at a frequency of 45 Hz and amplitude of 2 nm. The curves showed an increase in the required peak load from 18.43 to 26.74 mN together with a shift towards smaller indentation displacements by increasing the strain rate. This behavior indicated that the polymer displayed a harder response at the higher strain rates, which is in agreement with the nano-indentation results reported in the literature for an asphalt-filler mixture and for pure poly (methyl methacrylate) [34,35]. To further analyze the effect of strain rate on the response of the material in the load hold stage (before the unloading step), creep displacement was plotted as a function of strain rate, as shown in Figure 10. It can be clearly seen that a higher creep displacement was produced by performing the nano-indentation experiments at a higher strain rate indicating greater chances of creep occurrence at higher strain rates. A similar outcome for the effect of strain rate on the creep behavior was also reported in the literature for neat polyamide [36]. 

The nano-mechanical properties, including hardness and modulus, can also be measured for each strain rate and are depicted in Figure 11 and Figure 12 as a function of indentation displacement. At very small indentation displacements, large values of both modulus and hardness were evident (i.e., the indentation size effect). However, this effect was more noticeable for hardness, having values at smaller depths up to three orders of magnitude higher than the values at higher displacements. In the case of modulus, the difference in the values was not higher than even one order of magnitude. Moreover, the hardness also exhibited a strong strain rate dependency, a harder response at higher strain rates, while the dependency of the modulus on the strain rate was almost negligible. The rate dependency of hardness is realistic for polymers (i.e., visco-elastic-plastic materials) due to the direct relation between the hardness and the yield stress of the material. Conversely, the modulus is mainly linked with the Van der Waals interactions, which are not dependent on the strain or loading rate [37,38,39].

All the results presented above confirmed the existence of an indentation size effect in the investigated polymeric sample, as has also been reported in the literature for other polymeric materials [40,41]. However, it is not appropriate to justify the existence of an indentation size effect in polymers with the reasons used for metals, such as strain gradient plasticity, due to the larger elastic strain energy in polymers as compared to their viscous counterparts. Consequently, the indentation size effect in polymers may be linked with the Frank elasticity [42], i.e., the elastic strain resulting from the bending deformations of polymeric chains, and not with the plastic strain that is usually used to explain the indentation size effects in metals (which occur due to the permanent displacement of atoms) [42].

### 3.3. Analysis of Creep Behavior

In the normal indentation mode, the hardness and modulus of a polymer can be estimated by using the Oliver–Pharr method [21], which assumes pure elastic behavior of the material. However, polymers usually exhibit viscoelastic behavior and display a creep (time dependent) behavior. Therefore, this creep phenomenon has to be minimized for the successful application of the Oliver–Pharr method. For this purpose, the effect of peak load holding time on the creep behavior was analyzed by choosing different holding times of 0.1, 5, 10, 20, 30, 50, and 100 s with two constant peak loads of 30 and 100 mN using the normal mode of indentation (experiment # 5). The resultant load-displacement curves, shown in Figure 13 and Figure 14, displayed an increase in the creep depth with the increasing holding time. The results also revealed a similar shape for the loading and unloading curves while the starting point for the unloading curves were different for each holding time due to the different creep behavior under load. 

In order to clearly see the effect of holding time on the creep behavior, the creep displacement was plotted as a function of holding time for the 30 and 100 mN peak loads in Figure 15. The results displayed a significant increase in creep displacement (from 51 to 2553 nm in the case of the 30 mN load and from 107 to 3997 nm in the case of 100 mN) by increasing the holding time from 0.1 to 100 s. For the same holding time, a higher creep depth was evident for the higher indentation load. This creep behavior of UHMWPE was fitted with a logarithmic creep model, already reported in the literature [43,44,45] and given as follows:

(1) h=A·ln(B·t+1)

where t represents the holding time, h is the creep displacement, and A and B are the fitting parameters that represent the extent and rate of creep, respectively. The experimental creep data for our UHMWPE was fitted with the above-mentioned model using the fitting algorithms of Table Curve 2D software. The values of the model parameters for UHMWPE are presented in Table 1. Figure 15 displays quite good agreements between the model and the experimental data for UHMWPE for the two loads (R^2^ ≥ 0.98).

### 3.4. Effect of Holding Time on Hardness and Modulus

The hardness and elastic modulus of UHMWPE were also plotted as a function of holding time under load (Figure 16 and Figure 17). The results revealed a strong dependence of the nano-mechanical creep properties on the holding time, indicating a significant decrease in both hardness (from 47 to 19 MPa in the case of the 30 mN load and from 45 to 22 MPa in the case of the 100 mN) and modulus (from 1.16 to 0.69 GPa in the case of the 30 mN load and from 1.08 to 0.73 GPa in the case of 100 mN) by increasing the holding time from 0.1 to 100 s. This outcome is in agreement with the already reported results in the literature for poly(methyl methacrylate) by Jin et al. [46] for which a similar trend of modulus and hardness as a function of holding time was also displayed.

This decrease in mechanical properties of UHMWPE as a function of holding time may be linked with the creep deformation produced either due to the molecular chain mobility under stress or an imbalance between the stress and the deformation, i.e., the deformation not being proportional to the stress [47]. However, this creep effect would become negligible for longer holding times, resulting in a smaller recovery of modulus. Consequently, this provides the justification for the smaller modulus values at longer holding times (Figure 17). Furthermore, increasing the holding time also resulted in larger creep depths, resulting in higher indenter contact area, which eventually led towards the smaller hardness values (Figure 16). In the case of the higher applied load, a weaker dependency of mechanical properties on the holding time with smaller values of modulus and hardness were observed as compared to the response at smaller applied loads for times less than 30 s. This behavior may be associated with the extra creeping penetration, before the occurrence of creep relaxation, at higher applied loads [13]. 

### 3.5. Recommended Holding Time

Chudoba et al. [45] proposed a non-theoretically-based criterion to estimate the recommended holding time for a given material, in order to eliminate the creep problem, as follows: “*The holding period at maximum load has to be long enough such that the creep rate has decayed to a value where the depth increase in 1 min is less than 1% of the indentation depth*”. In order to use this criterion, the first derivative of logarithmic creep, obtained from Equation (1), was estimated to predict the recommended holding times for the two different loads. The obtained values of recommended holding times were 1183 and 908 s for 30 and 100 mN applied load, respectively (Table 2). Since the holding times calculated were much longer than the experimental ones, an extrapolation was made of the plot in Figure 15 (creep displacement as a function of holding time), to predict these holding times required to eliminate the creep problem.

The first derivative of the creep (i.e., creep rate) was also plotted as a function of holding time, as shown in Figure 18. This graph shows that for very small holding times the creep rate was very significant. However, the values of the creep rate decreased to very small values for the maximum calculated holding times of three orders of magnitude, i.e., 15–20 min. This recommended holding time can ensure the elimination of nose problems in nano-indentation experiments.

## 4. Conclusions

This study presents the effect of different experimental conditions on the nano-mechanical properties of UHMWPE obtained by using the normal and continuous stiffness modes of a nano-indenter. Various parameters, including amplitude, frequency, contact depth, strain rate, loading time, and peak load, were varied to investigate their influence on the resultant nano-mechanical properties of UHMWPE. The results showed a significant effect of amplitude, frequency, and strain rate on the hardness and modulus of the UHMWPE. Load-displacement curves showed a shift towards lower indentation depths along with an increase in peak load by increasing the indentation amplitude or strain rate. The results also revealed the strong dependence of hardness and modulus on the holding time. The experimental data of creep depth as a function of holding time was successfully fitted with a logarithmic creep model (R^2^ ≥ 0.98). In order to remove the creeping effect and the nose problem, recommended holding times (908 and 1183 s) were also proposed for the UHMWPE for the two applied loads (30 and 100 mN).

## Figures and Tables

**Table 1:** Values of fitting parameters of Equation (1) for UHMWPE.

Sr. #	Load (mN)	Parameter A (nm)	Parameter B (s^−^^1^)	R^2^
1	30	1134	0.08	0.98
2	100	1536	0.13	0.99

**Table 2:** Recommended holding times for UHMWPE for the two different peak loads.

Sr. #	Load (mN)	Recommended Holding Time (s)
1	30	1183
2	100	908
